# Hypertension and hypertension-related disease in mongolia; findings of a national knowledge, attitudes and practices study

**DOI:** 10.1186/1471-2458-13-194

**Published:** 2013-03-06

**Authors:** Alessandro R Demaio, Dugee Otgontuya, Maximilian de Courten, Ib C Bygbjerg, Palam Enkhtuya, Dan W Meyrowitsch, Janchiv Oyunbileg

**Affiliations:** 1 Copenhagen School of Global Health, University of Copenhagen, Øster Farimagsgade 5, DK-1014 Copenhagen, Denmark; 2 Public Health Institute, Mongolian Ministry of Health Olympic Street 2, Ulaanbaatar, Mongolia; 3 University of Copenhagen, Faculty Of Health Sciences, Øster Farimagsgade 5, DK-1014 Copenhagen, Denmark; 4 Section of Health Services Research Department of Public Health, University of Copenhagen, Øster Farimagsgade 5, DK-1014 Copenhagen, Denmark

**Keywords:** Hypertension, Blood-pressure, Non-communicable disease, Epidemiology, KAP, Mongolia, Asia

## Abstract

**Background:**

Mongolia has a high and increasing burden of hypertension and related disease, with cardiovascular diseases among the leading causes of death. Yet little is known about the knowledge, attitudes and practices of the Mongolian population with regards to blood pressure. With this in mind, a national Non-Communicable Diseases knowledge, attitudes and practices survey on blood pressure was implemented in late 2010. This paper reports on the findings of this research.

**Methods:**

Using a multi-stage, random cluster sampling method 3450 participant households were selected from across Mongolia. This survey was interviewer-administered and included demographic and socio-economic questions. Sample size was calculated using methods aligned with the World Health Organization STEPS surveys.

**Results:**

One fifth of participants reported having never heard the term ‘blood pressure’. This absence of health knowledge was significantly higher in men, and particularly younger men. The majority of participants recognised high blood pressure to be a threat to health, with a higher level of risk awareness among urban individuals. Education level and older age were generally associated with a heightened knowledge and risk perception. Roughly seven in ten participants were aware of the relationship between salt and blood pressure. Exploring barriers to screening, participants rated a ‘lack of perceived importance’ as the main deterring factor among fellow Mongolians and overall, participants perceived medication and exercise as the only interventions to be moderately effective at preventing high blood pressure.

**Conclusion:**

Rural populations; younger populations; men; and less educated populations, all with lower levels of knowledge and risk perception regarding hypertension, present those most vulnerable to it and the related health outcomes. This research intimates major health knowledge gaps in sub-populations within Mongolia, regarding health-risks related to hypertension.

## Background

Non-Communicable Diseases (NCDs) are now the leading causes of death in Mongolia and worldwide. Representing a growing threat to national and global health as well as social and economic development, these diseases are increasingly recognised by governments, non-government organisations (NGOs) and the scientific community as a chronic global epidemic. Their burden is expected to double by 2030 [1].

In Mongolia today, the issue of NCDs is building traction with government and the health sector. The statistics suggest a high and increasing burden of cardiovascular diseases and its associated risk factors. According to the 2009 World Health Organization (WHO) STEPS survey, 40% of Mongolians are estimated to be overweight or obese, 1 in 4 diagnosed with hyper-cholesterolaemia and almost 10% of males are now estimated to be diabetic [2]. With regard to blood pressure, hypertension prevalence was as high as 70% in older male populations and affects 1 in every 3 Mongolians aged 25–34 [2,3].

Hypertension is a risk factor for heart disease, peripheral vascular disease and cerebrovascular diseases. Conversely, hypertension may be induced by diabetes and chronic kidney disease. Recognised as a public health concern in Mongolia, its burden is rising - likely associated with the recent rapid epidemiological transition and associated dietary, socio-economic and lifestyle changes in this population [2,3]. Further, in the setting of rapid economic growth and urban migration, this burden is likely to grow in coming decades [4].Yet translating public health concern into policies and health-sector action has been stifled. In part, due to a lack of current, accurate, population-level knowledge, attitudes and practice (KAP) data.

KAP surveys have been widely used in public health since the 1960s and despite their usefulness in many epidemiological contexts, have attracted criticism as a stand-alone research tool [5]. Some also argue that their usefulness is limited in nations where levels of health knowledge have been characterised and more advanced research is now underway. But this is not so in Mongolia, where population-level health information remains unquantified despite large scale health interventions being currently implemented. Experiencing rapid increases in NCDs, policy and public health analysis continue to lack data around population level knowledge and attitudes, which in turn shape health interventions [6]. Simultaneously, populations with assumed inadequate knowledge and inappropriate attitudes and practices need to be identified before any intervention to address these change be implemented, including attempts at increasing population health-literacy [7]. The epidemiological frame currently guiding these projects has largely been biochemical and anthropometric burden of disease data collected via WHO STEPS surveys [8]. While crucially important, this has meant that little is known about the risk-perceptions, health priorities and knowledge of the populations regarding these health issues.

With this in mind, to provide rigorous, comparable and current KAP data for the triangulation of STEPS data, a national KAP survey on NCDs was implemented in Mongolia in late 2010 [9].

This paper is one in a series of publications reporting and exploring the knowledge, attitudes and practices of Mongolians with regards to NCDs through the use of the 2010 national NCD KAP survey data. This paper will focus on blood pressure, determinants of blood pressure and blood pressure screening, while others have explored diabetes; nutrition and alcohol KAPs. These papers aim to provide a context to established burden of disease data in order to better inform national and regional public health and health promotion policies and practices.

## Methods

### Setting and population

A door-to-door, household-based questionnaire was conducted on a nationally-representative sample. The full study protocol has been published [9]. Trained, Mongolian field workers from the National Institute for Public Health administered the questionnaire, which explored a range of NCD and risk factor domains, including hypertension.

### Sample size

A nationally-representative sample was estimated, reflecting the need for disaggregated data analysis according to gender, urbanicity and other key demographics. The calculation was estimated considering a 95% confidence level.

A design effect was adopted, based on methodologies used for the WHO STEPS to account for the sampling method used. Assuming a non-response rate of 10%, final sample size was estimated at 3,854.

### Sampling methods

Using a multi-stage, random cluster sampling method, participants were selected from individual households across Mongolia [9]. With 21 rural and urban sites each, chosen using proportional population to size (PPS) sampling. Sampling then used PPS methods again for secondary sampling units and simple random sampling from household lists for tertiary sampling units. Finally, a Kish method was used for participant selection within households [10].

While this survey does not sample the same exact participants as the 2009 WHO STEPS survey, its methodologies were aligned.

### Questionnaire

This KAP survey was an interviewer-administered, quantitative survey consisting of 102 questions, including demographic and socio-economic questions.

Knowledge was explored through a series of open and closed-ended questions. The first asked participants to self-rate their own perceived level of knowledge with regards to the concept of blood pressure. A four-level, ordinal-scale question, from no knowledge to very familiar, aimed to assess their own level of perceived awareness. After explaining the basic concept of blood pressure to all participants, a question then explored the relationship between blood pressure and health; probing for understanding that high blood pressure is related to health problems and disease. Further exploring this notion and to triangulate the findings’ accuracy, participants were then asked to identify the relationship between high blood pressure and specific body organ risk (brain, heart, eyes and kidneys).

In order to explore attitudes and practices, regarding blood pressure screening and prevention, participants were asked questions on their perception of the importance of screening attendance and programs. Further, they were asked to nominate the main reason they thought Mongolians may not attend screening, from a list of options.

With regards to prevention, exploring salt and its relationship with blood pressure, questions were posed on the relationship between dietary salt and blood pressure. These questions were aggregated to increase accuracy. Then, four prevention methods were verbally listed and participants were asked to rank their effectiveness on a three-point Likert scale from one or not effective, to three or highly effective. The mean of these, along with confidence intervals, were calculated.

Finally, regarding dietary salt, participants were asked whether they are conscious of the amount of salt they consume, that is, whether it is something they consider. They were then asked to choose the main source of dietary salt for Mongolians from a list of three possibilities.

### Quality control

In order to maximise scientific rigour in this research, a number of processes were included in the questionnaire methodologies. These included:

1. Translation and back translation

2. Peer and expert review

3. Pretesting using a Cognitive Interviewing [11]

4. Piloting.

### Ethical considerations

This study was conducted according to the principles of the Helsinki declaration. The Mongolian National Ministry of Health’s Medical Ethical Committee approved the study on the 06 October 2010.

Consent was implied if participants agreed to participate in the survey, once informed of the methods and use of data as well as its anonymous and confidential nature. Participation in this research was voluntary, and no financial remuneration was provided.

### Data analysis

Statistical Package for the Social Sciences (IBM SPSS 20.0.0 Statistics) was used for data entry and analysis. Data was weighted (sample and population weighting coefficients applied) to correct for differences between the sampled population and Mongolian census data. Complex sample descriptive rates and multivariate odds ratios were the main indexes used for analysis. Statistical significance was assumed at p <0.05 for all tests. Sampling error, with the potential to affect the accuracy of the results of this survey, was estimated from the standard error of variables, which were computed taking the cluster sampling technique into account.

This KAP survey was designed as a research tool to align and implement with, and triangulate the findings of the STEPS surveys. It was not possible to implement both surveys in 2010 and so 2009 STEPS data (for example, prevalence of hypertension) was used as a basis for interpreting the results of this KAP study.

## Results

In total, 3450 of the sampled 3,854 participants agreed to participate and were included in the analyses (89.5%), with one participant sampled from each household.

Almost 60% of participants were women, and half were from each rural and urban areas (Table 1).

**Table 1:** Descriptive information on sample population, disaggregated by age, sex, urbanicity, educational level and employment status

		**2010 National Census Data**	**n (% of Total)**
		**n (% of Total)**	
Total		1,843,285	3450 (100)
Gender	Male	891,146 (48.3)	1413 (42.0)
	Female	952,139 (51.6)	2037 (58.0)
Age (n=3450)	15-24	579,274 (31.4)	1100 (28)
	25-34	475,033 (25.7)	721 (24.3)
	35-44	387,541 (21.0)	630 (23.0)
	45-54	275,745 (15.0)	507 (19.2)
	55-64	125,692 (6.8)	492 (5.5)
Median Age		##	33 years
Location (n=3450)	Urban	##	1737 (50.3)
	Rural	##	1713 (49.7)
Education	Primary or less	##	219 (6.4)
(n= 3450)	Secondary School	##	2088 (60.5)
	Tertiary Schooling	##	1143 (33.1)
Employment (n=3425)	Student	##	717 (20.8)
	Employed	##	1503 (43.6)
	Unemployed	##	508 (14.7)
	Retired/Home	##	697 (20.2)

## Data not available. Source: Mongolian National Institute for Public Health.

The median age of participants was 33 years. Rural and urban median age was not significantly different (32 and 34 years, p = 0.08), while females, overall, were older than their male counterparts by three years (p <0.05).

The majority of participants were educated to a secondary school level with 33% having attended university, while 6.4% had received education to the level of primary or less. Students made up one-fifth of participants, of which half were women. More than 43% of participants reported to be employed and 14.7% currently unemployed. Retirees or home-makers accounted for 20% of the sample, of which 75% were women.

Comparing rural versus urban populations, no significant differences were found in terms of median age, gender distribution or employment status. Although, significantly more urban participants had received secondary or tertiary education than their rural counterparts (p <0.05).

### General Health Knowledge Regarding Blood Pressure

With respect to blood pressure, 17.4% (95%CI: 16.2% to 18.7%) of participants rated their knowledge as ‘never heard the term before’ – indicating no health knowledge with regards to blood pressure (Table 2). This basic health knowledge was significantly lower among younger populations, observed through both univariate and multivariate analyses; and male populations. These associations held after controlling for urbanicity, educational level and employment status. No significant difference in awareness was found between urban and rural populations, though there was a significant relationship between a lack of basic knowledge and lower levels of formal education. In addition, retirees and home-makers were the best informed with regards to blood pressure while the unemployed and students were significantly more likely to lack basic knowledge.

**Table 2:** Knowledge and attitudes toward blood pressure

		**Reported as ‘never heard ‘blood pressure’ before’**		**Perceived high blood pressure as a risk to health**		**Aware of three main organs at risk from BP**		**Blood pressure screening perceived as important**		**Aware that salt can affect and raise blood pressure**	
		**n (% of Total)**	MOR*	**n (% of Total)**	MOR*	**n (% of Total)**	MOR*	**n (% of Total)**	MOR*	**n (% of Total)**	MOR*
**Total** (3450)**		601 (17.4)		2701 (78.4)		1919 (54.2)		3327 (96.4)		2518 (71.1)	
**Gender**	Male (1413)	299 (21.2)	1.5 (1.2-1.8)	1047 (74.2)	1	689 (48.8)	1	1335 (94.5)	1	937 (66.3)	1
	Female (2037)	302 (14.9)	1	1654 (81.3)	1.4 (1.2-1.6)	1230 (60.4)	1.4 (1.2-1.7)	1992 (97.8)	2.5 (1.7-3.8)	1581 (77.6)	1.6 (1.1-2.3)
**Urbanicity**	Rural (1713)	275 (15.8)	1	1347 (77.7)	1	943 (54.3)	1	1658 (95.5)	1	1232 (70.9)	1
	Urban (1737)	326 (19.0)	1.1 (0.9-1.4)	1354 (79.0)	1.2 (1.1-1.5)	976 (57.0)	1.2 (1.1-1.4)	1669 (97.4)	1.8 (1.2-2.7)	1286 (75.1)	1.2 (1.1-1.4)
**Age**	15-24 (1100)	290 (26.9)	6.2 (3.9-9.8)	726 (67.3)	1	372 (34.5)	1	1010 (93.7)	1	657 (59.7)	1
	25-34 (721)	163 (22.5)	4.7 (3.1-7.0)	543 (74.9)	1.2 (0.9-1.6)	376 (51.9)	1.4 (0.8-2.6)	699 (96.4)	1.4 (0.8-.24)	492 (68.2)	1.03 (0.6-1.9)
	35-44 (630)	79 (12.4)	2.4 (1.7-3.4)	538 (84.2)	2.2 (1.6-3.0)	408 (63.8)	1.3 (0.7-2.3)	624 (97.7)	2.0 (0.9-3.9)	512 (81.3)	2.1 (1.0-4.1)
	45-54 (507)	38 (7.6)	1.1 (0.8-1.4)	444 (88.6)	3.4 (2.4-4.8)	372 (74.3)	1.7 (0.9-3.4)	492 (98.2)	2.6 (1.0-5.9)	412 (81.2)	2.2 (1.2-4.6)
	55-64 (492)	31 (6.1)	1	450 (88.8)	3.4 (2.3-5.0)	391 (77.1)	3.1 (1.3-7.4)	502 (99.0)	8.0 (2.1-17.4)	445 (90.4)	3.2 (1.4-7.0)
**Education**	Primary or less (219)	61 (27.9)	4.1 (2.6-6.3)	162 (74.0)	1	116 (53.0)	1	209 (95.4)	1	165 (75.3)	1
	Secondary School (2088)	444 (21.3)	1.8 (1.3-2.6)	1559 (74.7)	1.2 (0.9-1.7)	1047 (50.1)	1.1 (0.8-1.5)	2010 (96.3)	1.9 (1.0-4.0)	1448 (69.3)	0.9 (0.6-1.8)
	Tertiary (1143)	96 (8.4)	1	980 (85.9)	1.9 (1.3-2.9)	756 (66.1)	1.7 (1.2-2.3)	1108 (96.9)	1.6 (0.8-3.7)	903 (79.2)	1.3 (0.9-1.8)
**Employment**	Student (717)	186 (25.9)	2.2 (1.7-3.1)	478 (66.9)	1	224 (31.2)	1	667 (93.0)	1	443 (60.4)	1
	Employed (1503)	202 (13.4)	1.4 (1.0-1.8)	1230 (81.9)	1.2 (0.9-1.7)	923 (61.4)	1.5 (0.7-2.9)	1462 (97.3)	1.1 (0.5-2.4)	1129 (75.1)	1.8 (1.4-2.2)
	Unemployed (508)	124 (24.4)	2.5 (1.8-3.4)	383 (75.4)	0.9 (0.7-1.3)	274 (53.9)	1.5 (0.8-2.8)	491 (96.7)	1.3 (0.7-2.6)	363 (71.5)	1.5 (1.2-2.2)
	Home Maker/Retired (697)	80 (11.5)	1	595 (85.4)	1.9 (1.3-2.9)	486 (69.7)	1.7 (1.0-3.1)	682 (97.8)	1.7 (0.9-3.1)	576 (82.6)	2.2 (1.8-3.0)

* Multivariate odds ratios, adjusted for gender, urbanicity, age, educational level and employment status.

** Disaggregated sample sizes given may vary slightly for each question.

Two-fifths (40%, 38.3-41.5) of participants rated their knowledge as high and felt they were ‘very familiar’ with the concept of blood pressure. Women were more likely to rate their knowledge as high (42% versus 36%), as were tertiary graduated participants (46%, 42.9-48.7) and those currently employed (18.4% versus 11.0% in unemployed). All these differences were statistically significant. No significant differences were found between rural and urban groups.

Following this question, a brief explanation of ‘blood pressure’ was provided to all participants.

### Risk perceptions regarding high blood pressure and related disease

The majority of participants recognised high blood pressure to be a threat to health (Table 2). Females were significantly more likely to hold this view, 81.3% (95%CI: 79.6 to 83.0) versus 74.2 (95%CI: 71.9 to 76.5) in men. This was also significant when all other variables were controlled. Urban as well as older populations held higher risk perceptions (p <0.05). Education level was again linked to a heightened knowledge and risk perception (p <0.05).

Participants were then questioned on their awareness regarding the risk posed to specific body organs from high blood pressure, as another measure of risk perception and knowledge. This time, 54.2% were aware of the risks of blood pressure to the heart, kidneys and brain. Women were significantly more likely to be aware of the risks than men (MOR 1.4), and urban populations were again better informed than their rural counterparts (MOR 1.2). Tertiary educated, and oldest populations, were again most informed (p <0.05).

### Knowledge, attitudes and practices regarding blood pressure screening

Attitudes towards blood pressure screening were similar across all groups, once the concept of blood pressure was explained, with more than 95% (95%CI: 95.8% to 97.0%) of respondents perceiving screening programs to be valuable (Table 2). Small but significant differences were observed between men and women, 94.5% (95%CI: 93.3% to 95.7%) and 97.8% (95%CI: 97.2% to 98.4%). The oldest age group, aged 55 – 64 years, perceived the risk to be higher than younger age groups (p <0.05).

**Figure 1:**
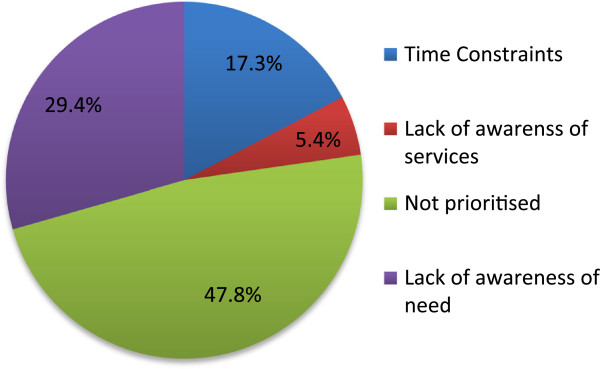
Perceived barriers to public blood pressure screening, total population.

Exploring barriers to blood pressure screening, participants rated a ‘lack of self-perceived importance’ as the main deterring factor for screening amongst fellow Mongolians (47.8%, 46.1-49.5) (Figure 1).

A ‘lack of awareness of the need’ to be screened was reported by almost three in ten responses (95%CI: 27.9-30.9). Time constraints, or ‘a lack of time’ was cited by 17.3% of Mongolians (95%CI: 15.4-19.2), while few (5.4%, 4.3-6.5) blamed a lack of awareness of screening services and access. No statistically significant differences were found between groups disaggregated by gender, urbanicity, educational experience or employment status.

Groups with less education tended to cite time constraints and a lack of awareness of the services, than more educated groups, though these trends were not statistically significant.

### Prevention of high blood pressure

Field surveyors then read participants a list of four commonly used prevention methods for high blood pressure and asked to weight their perceived effectiveness on a 3-point Likert scale from not effective to highly effective. Results between one and two represent low or no perceived-effectiveness, while results between two and three represent increasing perceived-effectiveness (Table 3).

**Table 3:** Perceived effectiveness of common prevention methods for high blood pressure, as measured by mean and associated 95% confidence-interval* from a three-point Likert Scale

		**Weight loss (Mean and 95%CI)**	**Dietary changes (Mean and 95%CI)**	**Medication (Mean and 95%CI)**	**Exercise (Mean and 95%CI)**
**Total**		1.85 (1.83-1.87)	1.90 (1.88-1.92)	2.16 (2.14-2.18)	2.04 (2.02-2.06)
**Gender**	Male	1.80 (1.77-1.83)	1.83 (1.8-1.86)	2.12 (2.1-2.14)	2.01 (1.98-2.04)
	Female	1.88 (1.86-1.9)	1.95 (1.93-1.97)	2.18 (2.16-2.2)	2.07 (2.05-2.09)
**Urbanicity**	Urban	1.92 (1.89-1.95)	1.93 (1.91-1.95)	2.21 (2.19-2.23)	2.08 (2.05-2.11)
	Rural	1.78 (1.73-1.75)	1.87 (1.82-1.84)	2.10 (2.08-2.1)	2.01 (1.98-2.01)
**Age**	15-24	1.7 (1.67-1.73)	1.82 (1.79-1.85)	2.08 (2.05-2.11)	1.99 (1.96-2.02)
	25-34	1.87 (1.83-1.91)	1.87 (1.83-1.91)	2.16 (2.13-2.19)	2.04 (2–2.08)
	35-44	1.94 (1.9-1.98)	1.97 (1.93-2.01)	2.2 (2.16-2.24)	2.07 (2.03-2.11)
	45-54	1.9 (1.86-1.94)	1.94 (1.9-1.98)	2.19 (2.15-2.23)	2.08 (2.03-2.13)
	55-64	1.95 (1.93-2.01)	1.98 (1.94-2.02)	2.23 (2.19-2.27)	2.09 (2.04-2.14)
**Education**	Less than Primary	1.70 (1.64-1.76)	1.72 (1.65-1.79)	2.08 (2.03-2.13)	1.91 (1.83-1.99)
	Secondary School	1.78 (1.76-1.8)	1.87 (1.85-1.89)	2.14 (2.12-2.16)	2.02 (2.0-2.04)
	Tertiary	2.00 (1.97-2.03)	1.99 (1.96-2.02)	2.21 (2.18-2.24)	2.12 (2.09-2.15)
**Employment**	Student	1.69 (1.65-1.73)	1.84 (1.8-1.88)	2.07 (2.04-2.1)	2.0 (1.96-2.04)
	Employed	1.92 (1.89-1.95)	1.94 (1.91-1.97)	2.18 (2.16-2.2)	2.08 (2.05-2.11)
	Unemployed	1.78 (1.73-1.83)	1.84 (1.79-1.89)	2.14 (2.1-2.18)	1.95 (1.9-2.0)
	H Maker/Retired	1.91 (1.87-1.95)	1.93 (1.89-1.97)	2.2 (2.17-2.23)	2.07 (2.03-2.11)

* Mean of less than 2 indicates a prevention method which is perceived to be ineffective.

Overall, participants perceived medication and exercise as the only interventions moderately effective at preventing high blood pressure. Medication was perceived to be significantly more effective than weight loss, dietary changes or exercise, with a mean of 2.16 (95%CI: 2.14-2.18). Exercise was perceived to be borderline effective with a mean score of 2.04 (95%CI: 2.02-2.06). Weight loss was perceived to be least effective of the four and on the whole, not effective with a score of 1.85. Dietary changes were also not perceived as effective in preventing high blood pressure and its complications.

Disaggregated analysis showed on average, women found all interventions to be more effective compared to men, as did urban populations compared to rural. These findings were all statistically significant (Table 3). The youngest populations perceived all prevention methods to be significantly less effective than older counterparts, with an increasing age-related gradient noted across all four prevention methods (all p <0.05). Across educational backgrounds, higher education was associated with a higher perceived effectiveness across all interventions with lower educated groups only reporting medications to be effective. Significant differences were also evident between employed and unemployed groups, with higher perceived prevention effectiveness among the employed, for three out of four methods.

### Knowledge, attitudes and practices regarding blood pressure risk factors

Turning to knowledge with regards to risk factors and focusing on dietary salt intake (Table 2), roughly seven in every ten participants were aware of the relationship between salt and blood pressure, both that intake affected blood pressure and that increased salt consumption could lead to a rise in blood pressure. Women were significantly more aware, with 77.6% of women recognising the link. Again, urban populations and older populations were significantly more informed with regards to this risk factor, independent of other factors. While students were comparatively less aware, only small differences were found between employed and unemployed groups. This level of awareness was not affected by educational background.

Questions were then posed exploring attitudes and knowledge around dietary salt and sources of dietary salt (Table 4). Participants were asked whether they were conscious of their salt consumption in their daily lives, to which two-thirds answered that they were (95%CI: 64.6%-67.8%). Females significantly more likely to be conscious of dietary salt intake and this difference was heightened by multivariate analysis. Advancing age (both univariate and multivariate models), employment and higher education were significantly associated with consciousness (p <0.05). No significant differences were found between urban and rural or educational groups.

**Table 4:** Perceptions and attitudes towards dietary salt and sources of dietary salt

		**Conscious of own dietary salt intake**		**Aware of processed foods as major source of dietary salt**	
		**n (% of Total)**	MOR*	**n (% of Total)**	MOR*
**Total**		2318 (66.2)		155 (4.5)	
**Gender**	Male	824 (58.4)	1.0	58 (4.1)	1.0
	Female	1494 (73.3)	1.6 (1.2-2.3)	97 (4.8)	1.2 (0.6-1.3)
**Urbanicity**	Urban	1181 (68.0)	1.0	61 (3.5)	1.0
	Rural	1137 (66.4)	1.0 (0.8-1.2)	94 (5.5)	1.6 (1.1-2.2)
**Age**	15-24	615 (57.1)	1.0	63 (5.8)	1.0
	25-34	478 (66.0)	1.0 (0.6-1.9)	32 (4.4)	1.1 (0.6-2.0)
	35-44	460 (72.0)	2.1 (1.0-4.2)	23 (3.6)	1.4 (0.8-2.6)
	45-54	370 (73.9)	2.3 (1.0-4.6)	16 (3.2)	1.7 (0.9-3.4)
	55-64	395 (77.9)	3.2 (1.4-7.0)	21 (4.1)	1.7 (0.9-3.4)
**Education**	Less than Primary	145 (66.2)	1.0	15 (6.8)	1.0
	Secondary School	1343 (64.4)	1.2 (0.7-2.3)	103 (4.9)	1.4 (0.8-2.5)
	Tertiary	830 (72.6)	3.1 (1.3-4.9)	37 (3.2)	1.8 (0.9-3.5)
**Employment**	Student	414 (57.7)	1.0	43 (6.0)	1.0
	Employed	1054 (70.2)	1.6 (1.3-2.0)	53 (3.5)	0.7 (0.4-1.5)
	Unemployed	320 (63.0)	1.2 (0.9-1.5)	22 (4.3)	1.2 (0.6-2.2)
	Home Maker/Retired	520 (74.6)	1.8 (1.2-2.3)	37 (5.3)	1.3 (0.7-2.3)

* Multivariate odds ratios, adjusted for gender, urbanicity, age, educational level and employment status.

Participants were then asked to choose the main perceived source of dietary salt, from either raw foods; added salt during cooking and eating; or salt consumed from processed meats, breads and biscuits. Nine in every ten participants (91.8%, 90.9-92.7) believed that the main source of dietary salt was that which they added to their meals during cooking or eating. Similar numbers of participants reported ‘raw foods such as milk, meat and vegetables’ and ‘processed foods such as bread, sausages and biscuits’ as their main sources, with four (3.7%, 3.1-4.3) and five percent (4.5%, 3.8-5.2) respectively. Disaggregated data showed no differences between sexes, age, educational or employment groups. While rural dwellers were statistically more likely to recognise the high dietary-salt contributions of processed foods to Mongolian diets, the two percent difference is unlikely to confer any meaningful population-level benefits.

## Discussion

In 2009, one third of sampled Mongolians suffered from hypertension, with 70% and 60% of Mongolian men and women aged 55–64 years affected respectively [2]. This prevalence represents a likely driver of heart disease, stroke and other chronic diseases. In addition, 60% of STEPS participants with hypertension were unaware of their condition at the time of screening, similar to US and UK prevalence from the 1960s-1970s [2,12,13]. This represents a group at great risk from the insidious diseases associated with raised blood pressure. The reasons for this lack of awareness, including knowledge of blood pressure, risk perceptions and barriers to screening, were previously unknown.

While most Mongolians are aware of the concept of blood pressure and have a broad understanding of its natural progression, 17% of Mongolians aged 15–64 had never heard the terms ‘blood pressure’ before. This echoed similar findings from India, where comprehensive, population-level knowledge is also lacking [14]. This lack of basic health knowledge was associated with male gender, younger age, lower levels of education and unemployment. Again, similar associations were observed in India [14]. Albeit not the same sample population as from the Mongolian STEPS survey, these groups were also those then identified with a higher risk or prevalence of hypertension. While many may argue that in high-income nations education may have a limited effect on behaviour change, limited health literacy has been associated with poor self-management of disease, reduced involvement in healthcare services and greater morbidity from chronic disease [7]. Basic health information has also been described as a precursor for health literacy and informed health decision-making [7]. Therefore, addressing this lack of basic health knowledge through health and education sectors could deliver gains in health and the mitigation of hypertension-related disease [15].

This is a need, though, which must be balanced against the potential harms of greater population-level health knowledge and associated anxiety, and the ethical implications of doing so in a resource-poor setting with continued limited access to treatments [2,15].

Knowledge of the determinants of blood pressure was high and awareness of the associations between weight, exercise and hypertension were also found. This knowledge was heavily skewed towards women, who were significantly more likely to understand these disease drivers. This finding may be linked to their roles as mothers and carers, or the fact that higher-education is more common amongst females [16,17]. Either way this represents a possible protective public-health factor, as mothers continue to be the main deciders of household diet and health seeking in Mongolia [16,18].

With regards to risk perceptions, 80% of Mongolians perceived high blood pressure as a health risk and almost all Mongolians perceived screening to be valuable. This understanding of risk is a valuable asset for health system responses [19]. In terms of shaping public health, this suggests that for those who are aware of the concept of blood pressure, there may be willingness for behaviour changes at the individual level and for policy changes at the community level.

Despite high knowledge and risk perceptions, previous studies show that almost half of the sampled population had never been screened for hypertension, reflected in the high-undiagnosed burden [2]. Exploring barriers to screening, Mongolians did not perceive screening as a priority. Although knowledge was adequate in much of the population and the risks understood, screening continues to not be prioritised by the sampled community. Therefore targeted campaigns, incentives or opportunistic screening may be more effective than current passive screening programs.

This low screening uptake seen in the STEPS survey could also be an outcome of the low perceived efficacy of prevention and treatment methods suggested by this KAP survey [2], That is, while Mongolians are aware of common prevention and treatment methods, these are perceived to be generally ineffective. Lifestyle-related changes for the prevention of hypertension were generally regarded as ineffective particularly amongst males who are also more likely to suffer from hypertension. Rural populations were significantly more likely to perceive medication and lifestyle changes as ineffective, possibly reflecting a health knowledge gap between rural and urban dwellers. This perceived lack of treatment efficacy along with low uptake of screening might go some way to explain the high prevalence of undiagnosed hypertension, and the low prevalence of antihypertensive therapy amongst Mongolians. The STEPS surveys found that two-thirds of Mongolians found to have high blood pressure were not medicated and 25% of those who were medicated were not adequately controlled or compliant [2]. Both of these figures were higher in men; up to three times, who also perceived medication to be least effective in this study.

Another example of a knowledge gap potentially leading to greater morbidity, is reflected in the very low level of understanding around dietary sources of salt. Mongolia is undergoing rapid economic development and urbanisation, associated with greater access to processed foods, higher in salt and saturated fats than traditional diet staples. Therefore the lack of understanding that these foods are high in salt and contribute a large proportion of dietary salt worldwide [20] presents a challenge to public health and agricultural food policy. With this in mind, regulatory responses such as package warnings or labelling and food education in schools may be appropriate.

### Limitations of research

It must be stressed that this KAP survey was not designed to be used as a stand-alone research tool for policy formation, as the limited impact of knowledge on health behaviours must be recognised [21]. This research was designed as a KAP tool to align and implement with, and triangulate the findings of the STEPS surveys – for more accurate interpretation and policy formation. The combined survey will be carried out in 2013 in Mongolia. Therefore, in this instance, data from the two, currently separate national surveys were triangulated where possible and some common conclusions drawn. As these surveys were not completed with the same sample populations though, merging of data and common regression analyses (for example KAP data with blood pressure prevalence) were not undertaken, as error due to sampling differences would likely affect the validity of results. In addition, the two surveys occurred one year apart. In a rapidly changing and transitioning nation, this would also represent differences in the sample and affect results.

Regarding selection bias and in order to minimise differences in selection of participants, key quality assurance processes were included in the training of interviewees, sampling methods and sampling size [9]. As a KAP survey questioning people’s values, attitudes, beliefs and knowledge, both recall bias and response bias must be acknowledged. Issues such as social desirability bias, particularly when discussing health, health knowledge and health behaviours would expect to impact on results [22,23]. In particular, such bias may work to mask the true scale of dangerous health behaviours or gaps in health knowledge and awareness. Measures were taken in question development as well as in the training of interview staff to ensure that these effects were minimised where possible.

Finally, an overrepresentation of females occurred despite the use of random sampling methods. This was due to greater compliance and a willingness among women to participate (Table 1). In order to account for this oversampling, data was weighted and all analyses saw disaggregation or adjustment by gender.

## Conclusions

This study highlights a number of areas of public health concern and identifies potential higher-risk populations with regards to blood-pressure-related disease. As Mongolia rapidly develops and continues to invest in its healthcare system, screening programs and prevention strategies [24], this study suggests that efforts to improve the health knowledge of younger Mongolians; men, especially in rural areas; and the less-educated, may be a valuable use of limited resources. In contrast to other settings, very basic health knowledge is lacking amongst these populations, information which is a precursor for health literacy and informed health decision-making [7]. The lacking of which, may stall current and future population-level health promotion efforts.

Findings also suggest that much of the Mongolian population understand the relevant health risks and are aware of main prevention and mitigation strategies, but perceive these to be unimportant or ineffective. This may make a case both for programs optimising opportunistic screening and regulatory changes such as around food labelling. But this research also intimates major health knowledge gaps in sub-populations within Mongolia, suggesting that public health responses which fail to include health education as a facet of their approach may be limited in their efficacy [25].

## Competing interests

The authors declare that they have no competing interests.

## Authors’ contributions

AD and DM drafted the manuscript. AD, OD, PA and MdC participated in the design of the study. OD obtained funding for the project. All authors read and commented on manuscript drafts.

## Pre-publication history

The pre-publication history for this paper can be accessed here:

http://www.biomedcentral.com/1471-2458/13/194/prepub
